# Airway remodeling heterogeneity in asthma and its relationship to disease outcomes

**DOI:** 10.3389/fphys.2023.1113100

**Published:** 2023-01-19

**Authors:** Aileen Hsieh, Najmeh Assadinia, Tillie-Louise Hackett

**Affiliations:** ^1^ Centre for Heart Lung Innovation, St. Paul’s Hospital, Vancouver, BC, Canada; ^2^ Department of Anesthesiology, Pharmacology and Therapeutics, University of British Columbia, Vancouver, BC, Canada

**Keywords:** asthma, airway remodeling, airway heterogeneity, computed tomgraphy (CT), magnetic resonance imaging (MRI), mucus plug, asthma therapeutics

## Abstract

Asthma affects an estimated 262 million people worldwide and caused over 461,000 deaths in 2019. The disease is characterized by chronic airway inflammation, reversible bronchoconstriction, and airway remodeling. Longitudinal studies have shown that current treatments for asthma (inhaled bronchodilators and corticosteroids) can reduce the frequency of exacerbations, but do not modify disease outcomes over time. Further, longitudinal studies in children to adulthood have shown that these treatments do not improve asthma severity or fixed airflow obstruction over time. In asthma, fixed airflow obstruction is caused by remodeling of the airway wall, but such airway remodeling also significantly contributes to airway closure during bronchoconstriction in acute asthmatic episodes. The goal of the current review is to understand what is known about the heterogeneity of airway remodeling in asthma and how this contributes to the disease process. We provide an overview of the existing knowledge on airway remodeling features observed in asthma, including loss of epithelial integrity, mucous cell metaplasia, extracellular matrix remodeling in both the airways and vessels, angiogenesis, and increased smooth muscle mass. While such studies have provided extensive knowledge on different aspects of airway remodeling, they have relied on biopsy sampling or pathological assessment of lungs from fatal asthma patients, which have limitations for understanding airway heterogeneity and the entire asthma syndrome. To further understand the heterogeneity of airway remodeling in asthma, we highlight the potential of *in vivo* imaging tools such as computed tomography and magnetic resonance imaging. Such volumetric imaging tools provide the opportunity to assess the heterogeneity of airway remodeling within the whole lung and have led to the novel identification of heterogenous gas trapping and mucus plugging as important predictors of patient outcomes. Lastly, we summarize the current knowledge of modification of airway remodeling with available asthma therapeutics to highlight the need for future studies that use *in vivo* imaging tools to assess airway remodeling outcomes.

## Introduction

Asthma affects an estimated 262 million people worldwide and caused over 461,000 deaths in 2019 (Vos et al., 2020). The disease is characterized by chronic airway inflammation, bronchial hyperresponsiveness (which form the targets of all pharmacological asthma therapeutics), and airway remodeling which involves all tissues of the airway wall (Holgate, 2008). Longitudinal studies have shown that current treatments for asthma (inhaled bronchodilators and corticosteroids) can reduce the frequency of exacerbations, but do not modify disease outcomes over time (Phelan et al., 2002; Sears et al., 2003). While patients can take precautions to avoid asthma-trigger exposure, inevitably 75% of patients report having asthma flare-ups and up to 45% regularly have a hard time breathing with day-to-day activities (The Lung Association, 2016). In this review, we emphasize the importance of studying the structural changes that occur with airway remodeling in asthma and how they relate to disease outcomes. We examine new evidence that has been generated using current *ex vivo* and *in vivo* imaging techniques to understand how heterogeneous airway remodeling influences airway closure and patient outcomes. Lastly, we review current therapeutics and their effect on features of airway remodeling.

### Asthma phenotypes

To further understand asthma pathology, the field has focused on clinical asthma phenotypes which have been generated from recognizable clusters of demographic, clinical, or pathophysiological characteristics of patients with asthma. Some of the most common asthma phenotypes are early-onset or late-onset allergic asthma, non-allergic asthma, smoking-related, obesity-related, or aspirin-exacerbated respiratory disease (AERD) (Kuruvilla et al., 2019). Most recently with immune profiling, the following Th2-high and Th2-low asthma endotypes have also been proposed. Within the Th2-high asthma endotype, there is higher airway eosinophilia, which is a good predictor of the patient’s responsiveness to inhaled corticosteroids (ten Brinke et al., 2004). Although it is clear that there is a strong relationship between Th2 cytokine levels, eosinophilic inflammation and asthma, the inflammatory response of the asthmatic individual is heterogeneous and not all individuals with asthma have eosinophilic inflammation. More recently, a systematic review showed that approximately 50% of asthma cases are Th2-low asthma, driven by Th1 and Th17 cells and neutrophilic inflammation (Douwes et al., 2002; Vock et al., 2010; Kuruvilla et al., 2019). Th2-low asthma is less sensitive or resistant to corticosteroid treatment due to the dysregulation of glucocorticoid receptors (Raundhal et al., 2015; Banuelos et al., 2017; Ramakrishnan et al., 2019; Al Heialy et al., 2020; Hong et al., 2022).

To date, endotyping the inflammatory profile within a patient with asthma has been important clinically to understand which patients will be the most likely to respond to inhaled corticosteroids or who will benefit from the treatment with biologics. However, independent of the asthma phenotype or endotype, the underlying pathologies of asthma are chronic inflammation, reversible bronchoconstriction, and airway remodeling. In asthma, airway remodeling of the conducting airway walls results in fixed airflow obstruction, but airway remodeling also significantly contributes to airway closure during bronchoconstriction during an acute asthmatic episode (Kuwano et al., 1993). Several longitudinal studies that have followed children to adulthood have shown that with increasing severity of the disease, fixed airflow obstruction is a persistent feature of the disease (Strachan et al., 1996; Phelan et al., 2002; Sears et al., 2003). Further, despite the use of inhaled corticosteroids and bronchodilators, it has been shown longitudinally that there is no improvement in fixed airflow obstruction over time if present in childhood (Strachan et al., 1996; Phelan et al., 2002; Sears et al., 2003). Thus, the goal of the current review is to understand what is known about the heterogeneity of airway remodeling in asthma and how this contributes to the disease process.

### Airway remodeling

**FIGURE 1:**
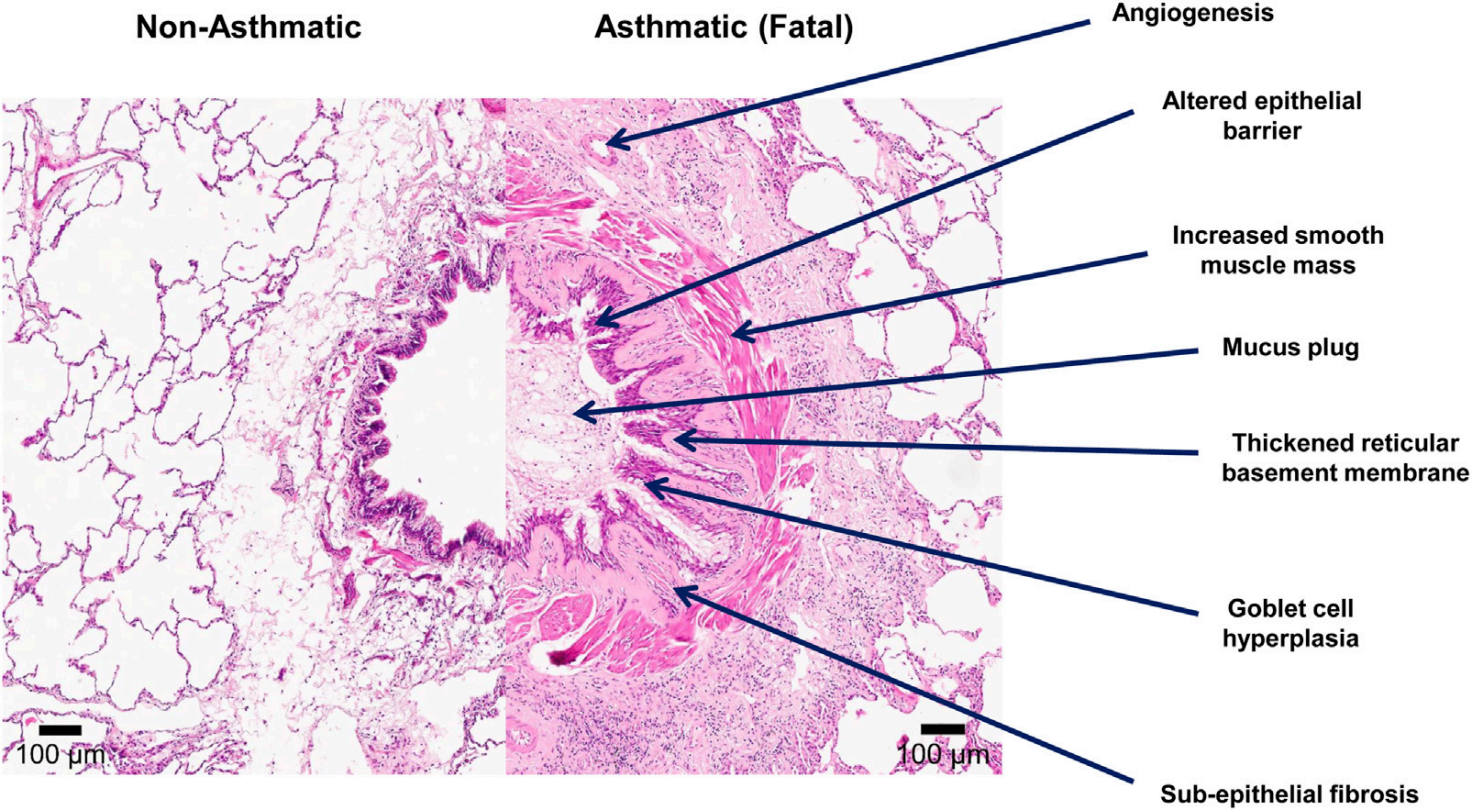
Features of airway remodeling in asthma. Lung tissue was formalin-fixed paraffin-embedded (FFPE) and stained with Hematoxylin and Eosin stains. The left image in the panel demonstrates a conducting airway from a non-asthmatic individual (11 years) with no history of respiratory disease. The right image demonstrates a conducting airway of a fatal asthmatic individual (15 years) showing features of airway remodeling including: an altered epithelial barrier with goblet cell hyperplasia, angiogenesis, increased smooth muscle mass, thickened reticular basement membrane, sub-epithelial fibrosis, and mucus plugging of the airway lumen.

Airway remodeling was first described in cases of fatal asthma by Huber and Koessler (1922). Airway remodeling in asthma refers to the structural changes that occur in both the large and small conducting airways and include loss of epithelial integrity, goblet cell and submucosal gland enlargement, basement membrane thickening, subepithelial fibrosis, increased smooth muscle mass, angiogenesis, and decreased cartilage integrity as highlighted in Figure 1 (Huber and Koessler, 1922; Haraguchi et al., 1999; Noble et al., 2002). Since then, features of airway remodeling have been documented for all stages of asthma severity and have been linked to reduced lung function, airway hyperresponsiveness, and the greater use of asthma medications (Holgate, 2008; Mostaço-Guidolin et al., 2019; Osei et al., 2020). The vast majority of what we understand about airway remodeling in asthma is derived from cross-sectional biopsy studies of the large conducting airways or post-mortem pathology on adult fatal asthmatics (Laitinen et al., 1985; Li and Wilson, 1997; Wilson and Li, 1997; Barbato et al., 2003; Payne et al., 2003; Tsartsali et al., 2011). It was previously proposed that chronic Th2 inflammation leads to a chronic cycle of injury resulting in airway remodeling over the lifetime of an individual with asthma (Mosmann et al., 1986; Le Gros et al., 1990; Metcalfe et al., 1997). However, recent studies have now shown that airway remodeling is not present at birth (Tsartsali et al., 2011), but features of remodeling are present by the age of 2–4 years of life, often before atopic inflammation is observed or a clinical diagnosis of asthma is made (Dunnill, 1960; Roche et al., 1989). Further, a systematic review of 39 studies examining the relationship between inflammation and airway remodeling concluded “Failure to demonstrate eosinophilic inflammation in children in the absence of airway remodeling is contrary to the hypothesis that inflammation causes these changes” (Castro-Rodriguez et al., 2018). Lastly, assuming airway remodeling is not reversible as is seen in rodent models, one would assume that airway remodeling would increase over time with persistent inflammation. However, Broekema et al. demonstrated in a large cohort of adult asthmatics with active asthma or a history of asthma, that independent of asthma status and medication use, the extent of airway remodeling and fixed airflow obstruction did not change over a 3-year time period (Broekema et al., 2011). Thus, the hypothesis that airway remodeling occurs over the lifetime of an asthmatic individual into adulthood in response to inflammation does not appear to be true. With that in mind, below we review the current knowledge of each airway remodeling feature associated with asthma.

#### Extracellular matrix remodeling

Abnormal thickening of the reticular basement membrane with increased deposition of extracellular matrix (ECM) proteins, fibronectin, collagen types I, III, and V, hyaluronan, laminin α2/β2, tenascin, and versican within the lamina reticularis is one of the hallmarks of airway remodeling in asthma and has been observed in both children and adults with mild to severe and fatal asthma in several histological studies (Roberts, 1995; Laitinen et al., 1997; Elias et al., 1999; Zeiger et al., 1999; Boulet et al., 2000; James et al., 2012a; Burgess et al., 2016). Several clinical studies have demonstrated that structural changes associated with reticular basement membrane thickening, correlate with airway hyperresponsiveness, suggesting abnormal ECM deposition is a fundamental abnormality involved in the pathogenesis of asthma (Ward et al., 2002; Tsurikisawa et al., 2010). More recently, Mostaço-Guidolin et al., using an automated unbiased approach involving the combination of colour segmentation to isolate airway wall features and then uniformly measuring the thickness distribution of each structure using Euclidean Distance Mapping, highlighted that not only is the reticular basement membrane thickened but, that there is greater variance or heterogeneity in the thickening of the reticular basement membrane (Mostaco-Guidolin et al., 2017). Specifically, the study highlighted the heterogeneity of reticular basement membrane remodeling both within a single airway wall and between patients, demonstrating airway remodeling is a dynamic process occurring at different levels or rates with any given airway within the lung (Mostaco-Guidolin et al., 2017). The study further showed that the basement membrane thickening and its heterogeneity in variance were not different between fatal and non-fatal asthmatics, irrespective of age and biological sex.

Increased deposition of collagen I, collagen III, and fibronectin have also been demonstrated in the lamina propria of patients with asthma (Roberts, 1995). Fibroblasts are the primary producer of ECM within the lung, and an increase in myofibroblast number within the lamina propria of conducting airways has been reported in asthmatic patients (Carroll et al., 2000; Boser et al., 2017). Myofibroblasts are ECM synthetic cells and have been suggested to be the cause of increased collagen I, collagen III, and fibronectin within the conducting airways of patients with asthma (James, 2005; Michalik et al., 2018). More recently, using second harmonic imaging, Mostaço-Guidolin et al. showed that the fibrillar collagen deposited within the lamina propria is not only overproduced but is highly disorganized and fragmented in both the large and small conducting airways in children and adults with asthma (Mostaço-Guidolin et al., 2019). The same study also showed that airway fibroblasts from asthmatic patients are defective at producing decorin, an important proteoglycan involved in collagen formation (Mostaço-Guidolin et al., 2019). As fragmented defective collagen can induce myofibroblast formation and stimulate increased ECM synthesis, it has been proposed that the lack of decorin production and disorganized fibrillar collagen formation may induce a feedback loop where fibroblasts are continually stimulated to produce more collagen resulting in a higher level of disorganized collagen and airway remodeling (Mostaço-Guidolin et al., 2019; Snelgrove and Patel, 2019).

Lastly, respiratory viruses may also play a role in increased ECM remodeling in asthma. Rhinovirus (RV) infection of airway smooth muscle cells increases the deposition of ECM proteins fibronectin, perlecan, and collagen IV (Kuo et al., 2011). Further, RV-induced ECM production also induced greater migration rates of airway smooth muscle on the ECM, suggesting that respiratory viruses can induce increased ECM deposition and increased airway smooth muscle mass from increased cell migration.

In summary, basement membrane thickening is an early and universal feature of airway wall remodeling in asthma. It has been proposed that basement membrane thickening may lead to loss of epithelial-fibroblast cross-talk leading to subepithelial fibrosis. Indeed Osei et al. have shown that epithelial-derived interleukin (IL)-1α is an important mediator in epithelial-fibroblast cross-talk and reduces inflammatory cytokine release and ECM production in healthy lung-derived fibroblasts (Osei et al., 2020). Whether the diffusion and activation of epithelial-derived factors such as IL-1α are altered due to ECM accumulation within the basement membrane in the asthmatic lung is still to be determined.

#### Airway epithelial remodeling

There is now strong evidence within the literature that impairment of epithelial barrier function in asthma is a key player in the disease pathogenesis through the initiation of airway inflammation and remodeling *via* the release of alarmins (e.g., TSLP, IL-25 and IL-33 expression) (Borish and Steinke, 2011; West et al., 2012; Paplińska-Goryca et al., 2018; Chan et al., 2019). Indeed gene variants in TSLP and IL33 are associated with increased expression of these epithelial alarmins (Gudbjartsson et al., 2009; He et al., 2009; Ferreira et al., 2011; Harada et al., 2011; Li et al., 2015; Shrine et al., 2019), and *ex vivo* studies have demonstrated higher expression levels of TSLP and IL-33 in airway epithelial cells derived from asthma patients cultured at air-liquid-interface (ALI) compared to healthy controls (Hackett et al., 2013). In response to environmental insults such as respiratory syncytial virus (RSV), particulate matter (PM) 10, or mechanical wounding, the airway epithelium of asthmatic patients has also been shown to be more sensitive compared to healthy controls, releasing greater levels of inflammatory cytokines including IL-6, IL-8 and granulocyte-macrophage colony-stimulating factor (GM-CSF), which are also known to be airway smooth muscle mitogens (Johnson et al., 2004; Hackett et al., 2011). Whereas, in response to rhinovirus infection, the bronchial epithelium of asthmatic patients has been shown to have a deficient innate immune response including decreased interferon-β expression, impaired apoptosis, and increased virus replication (Wark et al., 2005).

*In situ* studies have also revealed many structural changes within the airway epithelium of asthmatic patients, including disruption of tight junctions and adherens junctions, detachment of ciliated cells, increased numbers of goblet cells, basal cells (CK5+/p63+), and side population progenitor cells (Hackett and Knight, 2007; de Boer et al., 2008; Hackett et al., 2008; Knight et al., 2011; Xiao et al., 2011). In line with these findings, functional studies of airway epithelial cells derived from children and adults with asthma cultured at ALI have also demonstrated decreased expression of tight junction (increased permeability) and adherens junction molecules (e.g., E-cadherin, caveolin-1), and increased numbers of basal cells (Hackett et al., 2009; Hackett et al., 2011; Xiao et al., 2011). It has been shown that infections with respiratory viruses, such as RSV, disrupt epithelial tight junctions by activation of protein kinase D (Rezaee et al., 2013). Studies focused on RV infection have shown infection causes loss of tight junctions (ZO-1) in ALI-cultured airway epithelial cells from asthmatic children, which is more pronounced and sustained compared to airway epithelial cells from children with no asthma (Looi et al., 2016; Looi et al., 2018). A reduction in occludin expression in an NADPH-oxidase-dependent manner has also been implicated in RV infection, inducing barrier dysfunction (Comstock et al., 2011). In addition, RV infection in asthmatic subjects leads to less mitochondrial respiration compared to healthy control subjects, indicating that patients with asthma may have a less efficient immune response to virus infection (Huang et al., 2022). Disruption of airway epithelial barrier function in asthma is further supported by several genome-wide association studies (GWAS) that have found asthma risk genes and genetic loci associated with asthma (Cookson, 2004; Moheimani et al., 2016) that are involved in cell adhesion and airway epithelial barrier function such as *PCDH1* (protocadherin-1) (Koppelman et al., 2009), *ORMDL3* (orosomucoid-like 3) (Moffatt et al., 2007; Moffatt, 2008; Moffatt et al., 2010), *DPP10* (dipeptidyl peptidase 10) (Allen et al., 2003), *GPRA* (G protein-coupled receptor for asthma susceptibility) (Laitinen et al., 2004) and *CDHR3* (cadherin-related family member 3) (Bønnelykke et al., 2014; Basnet et al., 2019). *CDHR3*, which has been shown to be involved in cell adhesion and polarity, is the receptor for rhinovirus C, thus variants of this gene can modulate the susceptibility to infection (Bønnelykke et al., 2014; Li et al., 2015; Basnet et al., 2019). Although the mechanisms contributing to the loss of airway epithelial barrier function in asthma have not been fully elucidated, the thickening of the epithelium with increased numbers of basal cells may be a remodeling mechanism to support the attachment of the airway epithelium. This is supported by the early work of Evans and Plopper, who demonstrated that the reticular basement membrane is essential for anchoring the basal epithelial cells *via* hemidesmosomes and that within the conducting airways the height of the reticular basement membrane directly correlates with the height of the airway epithelium and number of basal cells (Evans and Plopper, 1988; Tsartsali et al., 2011). It is therefore not surprising that when measuring the thickness of the reticular basement membrane in asthma, Mostaço-Guidolin et al. showed that airway epithelial thickness directly correlates with reticular basement membrane thickness and that the two show great intra- and inter-subject heterogeneity (Mostaco-Guidolin et al., 2017).

Goblet cell hyperplasia (increased number of cells), metaplasia (change in cell phenotype) and mucus accumulation have been consistently reported in the conducting airways of patients with mild, moderate, severe, and fatal asthma (Kuyper et al., 2003; Wenzel et al., 2012; Malmström et al., 2017; Trejo Bittar et al., 2017; Elliot et al., 2018). In the epithelium of asthmatic patients, goblet cell metaplasia is the main contributor to increased numbers of goblet cells in the small airways rather than goblet cell hyperplasia (Curran and Cohn, 2010). Though there are many proposed mechanisms of goblet cell metaplasia, the main suggested mechanism is through trans-differentiation of club and ciliated cells into goblet cells, rather than the proliferation of pre-existing goblet cells (Lambrecht and Hammad, 2012). The second mechanism is through increased expression of inflammatory mediators which initiate goblet cell metaplasia (Curran and Cohn, 2010). Specifically, epidermal growth factor receptor (EGFR) and IL-13 induce both club and ciliated cells to transition into goblet cells through the transcription factors FoxA2, TTF-1, SPDEF, and GABA_A_R (deFelice et al., 2003; Whiting, 2003; Wan et al., 2004; Park et al., 2007; Xiang et al., 2007). It has also been proposed that goblet cell hyperplasia, metaplasia, and subsequent mucus over-production may result through epithelial compression during bronchoconstriction and airway hyperresponsiveness (Grainge et al., 2011).

Mucins secreted by goblet cells in the airway epithelium and submucosal glands are important for maintaining lung homeostasis by trapping particulate matter and pathogens, which are then removed from the lung *via* mucociliary clearance (Symmes et al., 2018). The primary mucins in the human airways are MUC5B and MUC5AC which are secreted throughout the small and large conducting airways, with MUC5B being the dominant secretory mucin in the healthy lung (Okuda et al., 2019). Increased MUC5AC expression has been observed by a number of asthma studies and mucus plugs have been shown to contribute to airflow obstruction in the conducting airways (Bonser and Erle, 2017). *MUC5AC* and *MUC5B* gene variants are also predicted to cause increased mucin production in asthma (Rousseau et al., 2007; Shrine et al., 2019). It has been hypothesized that the alterations in mucus composition and organization through changes in mucin gene expression as well as an increase in the number of goblet cells, can result in impaired mucus expectoration due to MUC5AC tethering and increased gel viscoelasticity (Bonser and Erle, 2017).

In summary, these studies have demonstrated that genetic defects or variations within the airway epithelium can cause, drive, or worsen asthma, leading to a defective epithelial barrier that can drive the disease process in response to interactions with the inhaled environment.

#### Angiogenesis

In 1960, Dunnill et al. first reported on bronchial artery dilation in lung tissue from 20 fatal asthmatic patients (Dunnill, 1960). Since then, it has been documented that an increased density of the bronchial vasculature (Vrugt et al., 2000; Wilson and Robertson, 2002; Tanaka et al., 2003; Barbato et al., 2006) leads to increased blood flow in both patients with asthma and animal models of the disease (Wanner et al., 1990; Csete et al., 1991; Kumar et al., 1998; Mendes et al., 2004), which further correlates with airflow limitation (Li and Wilson, 1997; Orsida et al., 1999; Vrugt et al., 2000), bronchial hyperresponsiveness (Orsida et al., 1999; Kanazawa et al., 2002a; Kanazawa et al., 2002b), and asthma severity (Hoshino et al., 2001a; Hashimoto et al., 2005; Chetta et al., 2007; Grigoraş et al., 2012). It has been proposed that the increased bronchial arterial angiogenesis in the airways of patients with asthma may promote the extravasation of inflammatory cells, the release of inflammatory mediators, and abnormal cell growth and proliferation leading to asthma pathology. More recently, the distal pulmonary arteries and veins of pediatric and adult asthma patients have also been shown to be remodeled with thickened walls resulting from increased deposition of disorganized, fragmented, and thicker collagen fibers, in comparison with control lungs (Mostaço-Guidolin et al., 2021). These data highlight that remodeling of the pulmonary vasculature occurs throughout the lung in asthma and is not restricted to the conducting airways which are the primary site of airway hyperresponsiveness and bronchoconstriction. Further, the deposition of disorganized and fragmented collagen fibers is a key feature of lung remodeling that occurs early in life, regardless of sex or disease severity to both the wall of conducting airways and the pulmonary vasculature.

In summary, future work is needed to understand the role of pulmonary vasculature remodeling in asthma and if it is a driver of the disease process or consequence.

#### Airway smooth muscle mass

Airway smooth muscle (ASM) hyperresponsiveness is a key feature of acute airflow limitation in asthma and its mechanisms of contraction and relaxation have been a subject of great investigation (Doeing and Solway, 2013; Wang et al., 2020). Increased ASM mass is a hallmark feature of airway wall remodeling in asthma which contributes to the narrowing of the airways and amplifies the effect of smooth muscle shortening (James et al., 1989). In 1993, Carroll et al. showed that the thickness of the ASM layer is increased in both large and small airways in patients with asthma, and there is more ASM mass with increasing disease severity (Carroll et al., 1993). In asthma, expansion of the ASM layer occurs through ASM cell hypertrophy (increased cell size), and hyperplasia (increased cell number) (Ebina et al., 1993; Araujo et al., 2008; James et al., 2012b). It has been proposed that hypertrophy and hyperplasia may be induced by the activation of elevated cytokines in the asthmatic lung, including TGF-β1, EGF, platelet-derived growth factor (PDGF) (Hirst et al., 1996; Panettieri et al., 1996; Cohen et al., 2000; Chen and Khalil, 2006). Additionally, ASM cells derived from asthmatic patients *in vitro* have been shown to have a significantly higher rate of proliferation compared to ASM cells derived from healthy individuals, which was shown to occur due to the dysregulation of C/EBPα, a transcription factor responsible for inhibition of proliferation through the glucocorticoid receptor (Johnson et al., 2001; Roth et al., 2004). Further, ASM cells from asthmatic patients have increased mitochondrial biogenesis and increased fatty acid consumption, which is thought to drive increased ASM cell proliferation in asthma (Esteves et al., 2021). These changes in ASM mass lead to mechanical changes in the cells ability to relax, contract, and lengthen, which play important roles in luminal narrowing and airflow restriction (Seow et al., 1998; Doeing and Solway, 2013). Lambert and Wiggs established that ASM contraction and consequently, airway narrowing can result in airway mucosal folding (Lambert, 1991; Lambert et al., 1994; Wiggs et al., 1997). This not only provides significant airway stiffness but also significant load against the ASM contraction, particularly in conjunction with the thickening of the airway wall and alterations in ECM deposition (Lambert, 1991; Lambert et al., 1994; Wiggs et al., 1997). During inspiration, radial tethering of the alveolar walls stretches the conducting airways and vessels open. However, in asthma, increased ASM mass causes the ASM to shorten excessively against the elastic loads provided by the lung parenchyma, parallel airways and vessels, and thus results in mucosal folding (Seow et al., 1998). Johnson et al. showed that when human airway epithelial cells are mechanically compressed, mimicking bronchoconstriction, this results in the secretion of ASM cell mitogens which include IL-6, IL-8, monocyte chemoattractant protein-1, and matrix metalloproteinase (MMP)-9 (Johnson et al., 2004). The deformed epithelium also released endothelin-1, which can augment both the basal tone and histamine-induced contraction of ASM cells. Moreover, it has been shown that repeated airway bronchoconstriction leads to higher expression of epithelium-generated TGF-β, which may lead to thickening of the sub-epithelial collagen layer (Grainge et al., 2011). This suggests that bronchoconstriction due to ASM contraction plays a role in inducing basement membrane thickening (Noble et al., 2014). Together, these studies suggest that independent of allergic inflammation, ASM bronchoconstriction and deformation of the airway epithelium may be sufficient for inducing features of airway wall remodeling and asthma progression (Camoretti-Mercado and Lockey, 2021).

It has been previously shown that placing a load on healthy airways stretches the ASM more than the optimal length, causing them to produce less contractile force (Seow et al., 1998). However, as we now know that airways from asthmatic subjects produce significantly greater force, solely an increased amount in ASM mass cannot be responsible for the amount of force produced, which suggests there may be alterations in either the contractile ability of myocytes or myocyte interactions (Brusasco et al., 1998; Seow et al., 1998). Chin et al. demonstrated that the mechanical properties of human tracheal ASM from asthmatic and non-asthmatic subjects were comparable except for increased passive stiffness and attenuated decline in force generation after an oscillatory perturbation (Chin et al., 2012). The authors concluded that these findings may explain the reduced bronchoconstriction induced after a deep inspiration in asthmatic subjects. More recently Ijpma et al. have shown that human ASM from asthma subjects is hyperreactive only in intrapulmonary airways and not in the trachea, suggesting ASM hypersensitivity is dependent on the airway generation within the lung (Ijpma et al., 2020).

More recently the role of ASM in asthma pathology has been shown to extend beyond bronchoconstriction. There is now compelling evidence that ASM is involved in airway inflammation through the release of inflammatory chemokines IL-1β, TNFα or immunomodulators including IL-5, IL-13, TGF-β (Hakonarson et al., 1999; Coutts et al., 2001; Moynihan et al., 2008). ASM additionally releases eotaxin, increasing recruitment of eosinophils, and immune cells such as T-lymphocytes and mass cells have been shown to infiltrate and accumulate within the ASM layer (Ammit et al., 1997; Ghaffar et al., 1999; Moore et al., 2002; Berger et al., 2005; Ramos-Barbón et al., 2010). ASM can also regulate ECM composition through the secretion of matrix metalloproteinase (MMP)-9 and MMP-12 (Araujo et al., 2008). Further, ASM cells have been shown to secrete more collagen I and perlecan, and have decreased laminin α1 and collagen IV expression (Johnson et al., 2004). Additionally, Esteves et al. have recently shown that ASM from asthmatic patients increases RV replication in the epithelium after infection by secreting CCL20, inhibiting the epithelial protein kinase RNA-activated antiviral pathway (Esteves et al., 2022).

In summary, the contribution of ASM in asthma is multifactorial and beyond the effects of bronchoconstriction alone. ASM contraction not only alters the mechanical properties of the airway wall but also contributes to airway remodeling which influences other structural cells of the airway (epithelial, fibroblasts and vasculature) and immune modulation (inflammatory cells).

### Heterogeneity of lung ventilation and airway remodeling

While the histological studies described above have provided extensive knowledge on different aspects of airway remodeling in asthma, they have relied on biopsy sampling or autopsy lungs which have several limitations to note. Biopsy samples are limited in the airway wall structures that can be sampled which are primarily the epithelium, basement membrane and laminar reticularis, and the tissue artefacts that can be induced when crunched by the biopsy forceps. Additionally, lung biopsies primarily allow for the larger airways to be sampled and it is the branch point of two daughter airway generations that is sampled which can make sampling of the airway wall very difficult. Lastly, it is very difficult to sample multiple airway generations to assess the heterogeneity of airway remodeling within the lung of asthmatic patients. Autopsy studies on lungs from fatal asthmatics offer a unique opportunity to assess the heterogeneity of airway remodeling within multiple airway generations and across lung height, however, it does not provide the opportunity to assess the spectrum of the asthma syndrome specifically mild and moderate asthma. Below we describe the various approaches that have been used to assess the heterogeneity of airway remodeling and its effects on lung function. The inherent heterogeneity of lung ventilation has long been of great interest, dating back to 1956 when the uneven distribution of ventilation was hypothesized to influence the mechanical properties of the dynamic lung (Otis et al., 1956). This hypothesis was first explored using rat and canine models, which revealed that exposure to methacholine or histamine resulted in bronchial responsiveness that was heterogeneous in individual airways (Ludwig et al., 1987; Saldiva et al., 1992). This finding of heterogenous airway responsiveness was confirmed in human lung explants in 1997 by Minshall et al. who used agarose-filled lungs that were cross-sectioned and stimulated with methacholine (Minshall et al., 1997). To understand ventilation heterogeneity, many computational models have since been developed but many have assumed a simplified airway tree structure that did not provide an accurate model of the human lung (Neelakantan et al., 2022). In 1997, Bates and Thorpe developed a computational model which accounted for asymmetric branching patterns based on Horsfield and Cummings’s data (Horsfield and Cumming, 1968) on the branching of the human lung and stochastic heterogeneity (Thorpe and Bates, 1997). This model demonstrated that random heterogeneous airway narrowing in a human lung can lead to increased lung impedance (Kuwano et al., 1993). Though heterogeneous bronchoconstriction throughout the airway tree was well-established, the question remained if this occurs to the same or greater magnitude in diseased lungs. To answer this question, in 1999 Gillis and Lutchen used a computational lung model to show that when the same heterogeneous airway smooth muscle shortening is applied to a healthy lung and asthmatic lung, the healthy lung experiences mild changes but in the asthmatic lung, this would lead to greater changes in lung resistance (Gillis and Lutchen, 1999). This work demonstrated that airways within the lung of a patient with asthma are predisposed to heterogeneous bronchoconstriction. These data highlight that in asthma, the disease process is not occurring uniformly across the lung and may have spatial and temporal differences. Importantly, it has also been shown that in asthma, baseline ventilation heterogeneity of the conducting airways can predict airway hyperresponsiveness, independent of airway inflammation (Downie et al., 2007). More recently, Pascoe et al. demonstrated heterogeneous remodeling of randomly selected small airways within the lungs patients with fatal and non-fatal asthma compared to donor control lungs (Pascoe et al., 2017). Using their histological measurements of airway remodeling in a computational model of airway narrowing, they showed that heterogeneous airway remodeling can lead to increased airway closure (Pascoe et al., 2017). Direct comparisons have also been made between daughter airways bifurcating from a common parent airway and shown great heterogeneity in parallel daughter airways in response to inhaled methacholine (Carroll et al., 2015). These findings have led to the hypothesis that an individual airway’s remodeling phenotype is determined by its own mechanical and biological properties and is less influenced by its surrounding environment (Carroll et al., 2015). However more recently, Pascoe et al. have developed a mathematical model to demonstrate that remodeled airways are spatially correlated, which may be caused by cycles of bronchoconstriction and mechanotransduction (Pascoe et al., 2020). The study further confirmed this finding in human subjects with and without asthma (Pascoe et al., 2020). These results demonstrate the importance of understanding heterogeneous airway remodeling and its spatial distribution which may be responsible for ventilation heterogeneities in asthma.

### *In Vivo* imaging

The use of computed tomography (CT) scanning and magnetic resonance imaging (MRI) has enabled the 3-dimensional (3D) assessment of the lung structure *in vivo* in patients with asthma. This has overcome many limitations of histological studies which could only be conducted on lungs from fatal asthma patients, and the lack of airway wall structures sampled in an airway wall biopsy sample. Over the past three decades, numerous studies have measured airway remodeling and air trapping in patients with asthma using both qualitative, semi-quantitative, and quantitative CT/MRI techniques. Gupta and colleagues were the first to describe increased bronchial wall thickening in patients with severe asthma (*n* = 185) using CT (Gupta et al., 2009). Later, their group and other research groups, showed using quantitative measures including luminal volume and airway wall volume, that the large airways (measurements able to be obtained up to 5–6th generation of airways) (Brillet et al., 2007; Gupta et al., 2014; Grenier et al., 2016) are not only remodeled in patients with severe disease but are also remodeled in patients with mild/moderate asthma (Niimi et al., 2000; Brillet et al., 2013). Prior research has also demonstrated using CT that following methacholine-induced bronchoconstriction, the large conducting airways (>2 mm diameter) narrow more heterogeneously and have a larger decrease in airway lumen area in asthmatic patients compared to control patients (King et al., 2004). CT imaging has also enabled the assessment of disease progression over time within patients. In a longitudinal study, Witt et al. showed that severe asthma subjects have a greater decline in postbronchodilator FEV_1_% over time compared to those with mild-to-moderate asthma, which inversely correlated with significantly increased wall area percent and wall thickness percent in multiple airway generations, demonstrating the association between lung function loss and airway remodeling over time (Witt et al., 2014). This finding was also confirmed by Awadh et al. who showed that the wall thickness of large conducting airways is associated with lung function loss and disease severity (Awadh et al., 1998).

Several studies have also tried to correlate measures of large airway wall remodeling on CT with histopathological changes within the airway wall. Aysola et al. demonstrated that airway wall thickness at the third generation, correlated with increased epithelial and reticular basement membrane thickness on histology (Aysola et al., 2008). However, Kaminska et al. showed that airway wall thickness did not significantly correlate with the histopathological features of remodeling, including epithelial detachment, airway smooth muscle area, basement membrane thickness, and submucosal fibrosis, observed on biopsy (Kaminska et al., 2009). These findings indicate that there may be large variability in the degree of airway remodeling amongst patients with asthma that is detectable by CT imaging. Indeed, both studies showed variability in airway morphology between airways in the same patients and within-subject groups, which further highlights the need for assessment of the heterogeneity of airway wall remodeling in asthma.

### *In Vivo* imaging of ventilation defects in asthma

Several studies have used expiratory CT scans to analyze areas of low attenuation to identify areas of air trapping in asthmatic subjects (Newman et al., 1994; Stern and Frank, 1994). Air trapping is the retention of excess air within the lung after expiration, usually in distal parts of the lung, which often correlates with airflow limitation (Arakawa et al., 1998). Several methods have been used to quantify gas trapping including the ratio between mean inspiratory and expiratory lung attenuation (E/I) (Gono et al., 2003), lung density < 900 Houndsfield units (HU) at full expiration (Newman et al., 1994), lung density < −900 HU at full expiration (Busacker et al., 2009), and the difference between inspiratory and expiratory attenuation (Tunon-de-Lara et al., 2007). Using image registration, on lung CT scans at multiple volumes, Choi et al. have shown that severe asthmatic subjects not only have reduced air volume, but that air trapping occurs in the lower lobes more compared to the upper lobes (Choi et al., 2013). Using expiratory CT lung scans from the Severe Asthma Research Program (SARP) cohort, Busacker et al. performed a multivariate analysis demonstrating that subjects with air trapping were more likely to have a history of asthma-induced hospitalizations, and longer disease duration, suggesting that air trapping can identify individuals with high risk for a severe disease phenotype (Busacker et al., 2009). Further, Gono et al. have demonstrated that asthmatic subjects with persistent lung function loss, have greater ratios of airway wall thickness to total diameter and percentage wall area of the right bronchus, which correlated with more air-trapping compared to the asthmatic subjects with normal spirometry after bronchodilator inhalation (Gono et al., 2003). Together these studies suggest that increased airway remodeling contributes to air trapping.

**FIGURE 2:**
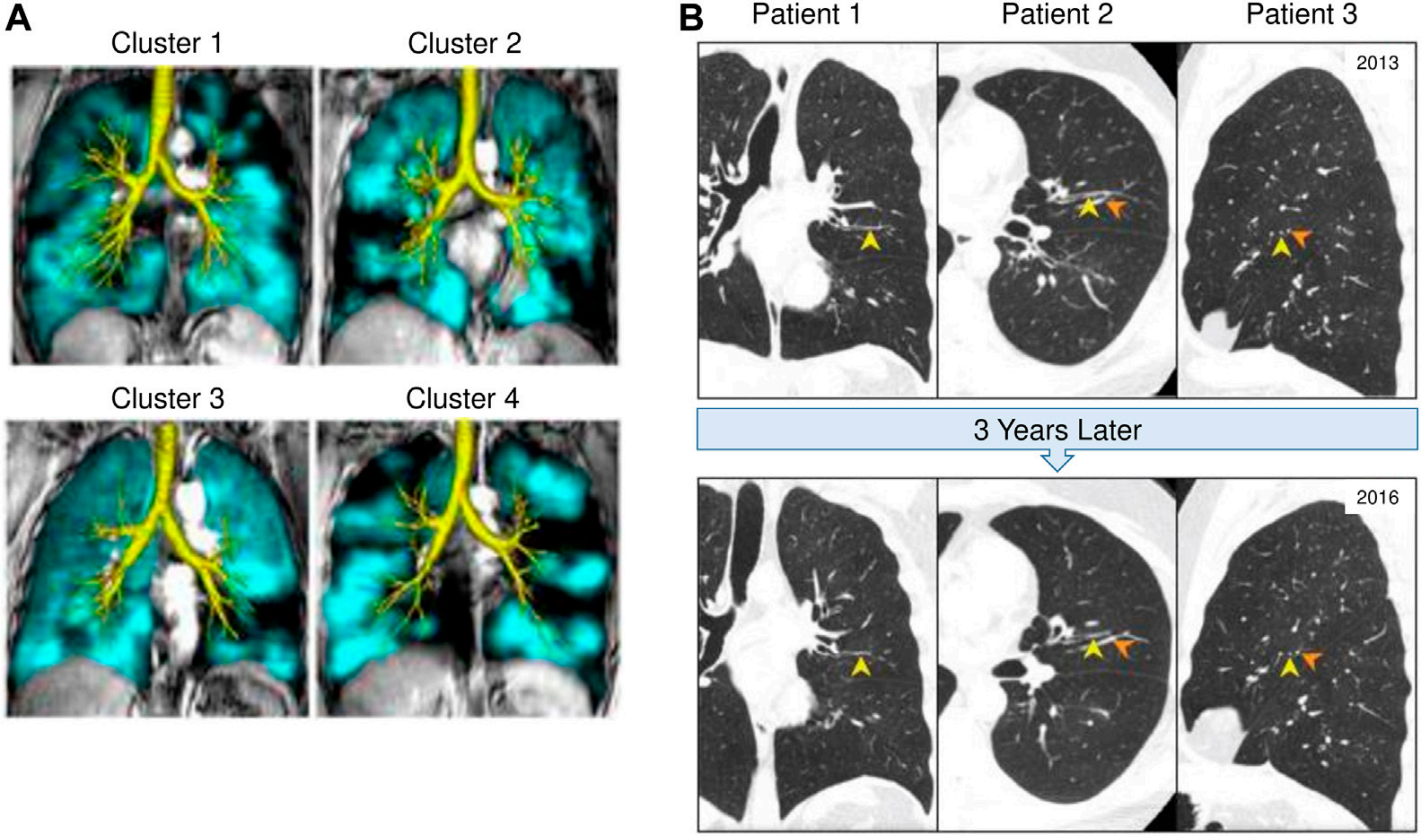
*In vivo* imaging of mucus plugging and ventilation defects in CT and MRI. **(A)** Lung computed tomography (CT) scans from three different asthma patients (A1, A2, and A3), which demonstrate the persistence of mucus plugs (Yellow arrowheads) in the same airway at baseline (2013) and 3 years later (2016). Orange arrowheads indicate blood vessels. This figure was adapted with permission from Figure 1 by Tang M et al. published in the American Journal of Respiratory and Critical Care Medicine 2022. **(B)** ^129^Xe magnetic resonance imaging (MRI) ventilation (teal) and computed tomography (CT) airway tree (yellow) for representative participants from four different asthma clusters. Cluster 1 has moderate heterogeneity as seen on MRI, moderate wall thickening and minimal luminal narrowing on CT. Clinically, cluster 1 has normal obstruction and no gas trapping. Cluster 2 has moderate MRI heterogeneity, significant wall thickening and minimal luminal narrowing. Clinically, this cluster pattern appears in females only, with moderate obstruction and gas trapping. Cluster 3 has moderate MRI heterogeneity with moderate wall thickening and significant luminal narrowing. Clinically, they tend to have more severe asthma and moderate obstruction with minimal gas trapping. Cluster 4 has significant MRI heterogeneity and moderate wall thickening with significant luminal narrowing. Clinically, cluster 4 patterning is male dominant with severe gas trapping and obstruction. Cluster analysis of asthma using MRI in combination with CT imaging may provide useful insight for asthma phenotyping and treatment decisions. This figure is adapted with permission from Figure 3B by Eddy R et al. published in Journal of Magnetic Resonance Imaging 2022. Copyright from RightsLink/John Wiley and Sons.

MRI with hyperpolarized gas such as Helium or Xenon has been used as a non-invasive technology to provide regional information on ventilation distribution within the human lung (Albert et al., 1994; Bachert et al., 1996; Kern and Vogel-Claussen, 2018). In asthma, MRI has shown that ventilation defects persist in the same location of the lung with repeated bronchoconstriction, demonstrating that areas of airflow obstruction tend to re-occur in the same airways (de Lange et al., 2007). De Lange et al. have also shown that patients with severe asthma have greater ventilation defects detected on MRI compared to those with mild to moderate asthma (de Lange et al., 2007). Interestingly, MRI has revealed ventilation defects exist even in subjects who have asymptomatic asthma with normal spirometry (Altes et al., 2001). This suggests that airflow obstruction and air trapping are occurring at a level that cannot yet be detected by spirometry. Altes et al. have shown that lung ventilation defects observed using MRI correlated with several clinical features of asthma including severity, medication use, decreased lung function (FEV_1_/FVC), and blood eosinophils (Altes et al., 2016). CT imaging is often used in conjunction with MRI to assess the lung structure in relation to the functionality of the lung. More recently Eddy et al. have combined MRI and CT imaging to identify phenotypic clusters which may have different clinical outcomes as demonstrated in Figure 2A (Eddy et al., 2022). These studies have important implications for how *in vivo* imaging can be used to support treatment decisions. Importantly, the same ventilation defects that have been observed on hyperpolarized gas MRI images in asthma, correspond to the same regions of air trapping that can be identified on expiratory CT scans (Fain et al., 2008).

### Mucus plugging

More recently CT has been used to visualize and quantify the presence of mucus plugs in asthmatic patients. In 2018, Dunican et al. analyzed CT scans of 146 adults with asthma and 22 healthy controls in the SARP cohort and established a method of scoring mucus plugging on CT scans based on a bronchopulmonary segment-based scoring system to quantify mucus plugging (Dunican et al., 2018). They showed that 67% of asthmatic subjects with FEV_1_ less than 60%, had a high mucus score (defined as mucus plugs present in more than four segments) which was also associated with increased sputum eosinophils and eosinophil peroxidase (EPO) levels (Dunican et al., 2018). Subsequently, a longitudinal study by Tang et al. assessed mucus plugs longitudinally using CT scans from the SARP cohort and found that 82% of subjects with mucus plugs at baseline still had mucus plugs at the third-year follow-up scan. Moreover, 65% of visible mucus plugs remained in the same airway segment as demonstrated in Figure 2B (Tang et al., 2022). These data demonstrate that chronic mucus plugs persist over time which emphasizes the need to clear mucus plugs to improve lung function in severe asthma patients (Tang et al., 2022). To evaluate the localization of mucus plugs, Yoshida et al. used a curved, multiplanar reconstruction (MPR) technique, a newly developed technique which provides greater accuracy and visualization than standard CT images (Yoshida et al., 2020). This study showed that when subjects were in the stable phase of asthma, the fourth and fifth-generation bronchi in the lower lobes had a greater frequency of mucus plugs. However, when subjects with asthma were undergoing an exacerbation, the upper lobes had a higher frequency of mucus plus in the fifth and sixth generation bronchi. In addition, Yoshida et al. showed that over 40% of conducting airways of the lower lobes were obstructed by mucus plugs (Yoshida et al., 2020). Lastly, a study involving 44 patients with severe asthma from the SARP cohort, assessed mucus plugging on CT scans, and showed that in the same bronchopulmonary segment where there was a mucus plug present, there were greater ventilation defects (Mummy et al., 2022). Together, these studies suggest that airway remodeling and mucus plugging are important causes of the heterogenous ventilation defects in asthma. It will be important to determine if remodeled airways are associated with mucus plugs and the key cells and mediators driving this process.

In summary, CT and MRI imaging are providing great insight into the heterogeneity of airway remodeling within the asthmatic lung and mucus plugs are chronic, persistent, and prevalent thus there is an urgent need to target mucus plugs to improve air trapping in asthma. Unfortunately, one remaining limitation of CT imaging apart from radiation exposure is the resolution to assess the airway tree at the cellular level to understand the disease pathology. The current resolution of CT (.85–1 mm in diameter) (Hasegawa et al., 2006; Kurashima et al., 2013; Kirby et al., 2018), depending on the radiation dose used does not enable the visualization of the smallest conducting airways (<2 mm in diameter) (McDonough et al., 2011), which are thought to contribute to the greatest impact to airway closure during bronchoconstriction and fixed airflow obstruction (airway remodeling) (Kurashima et al., 2013). More recently, microCT with a spatial resolution of up to 1 μm has enabled volumetric imaging and quantitative assessment of the small conducting airways, respiratory airways, and alveoli (Haefeli-Bleuer and Weibel, 1988; McDonough et al., 2011). To date, microCT has been applied to assess the morphometry of the normal human lung and how it is modified in the pathology of chronic obstructive pulmonary disease (COPD) (McDonough, 2012; Koo et al., 2018), lung cancer (Holbrook et al., 2021), Idiopathic Pulmonary Fibrosis (IPF) (Khalajzeyqami et al., 2022), cystic fibrosis (CF) (Wielpütz et al., 2011), and bronchiectasis (Wurnig et al., 2014). However one limitation of microCT is that it cannot be used *in vivo* in humans due to the small field of view and higher radiation dose. To date, research on asthma using microCT has been limited to murine models of asthma (Shofer et al., 2007; Lederlin et al., 2012; Paik et al., 2014). These studies have shown that it is possible to monitor airway remodeling (wall thickness, epithelial hyperplasia and smooth muscle hypertrophy) non-invasively in asthmatic mice which may be important for future drug testing. Future work using microCT to assess human samples will be important to bridge the resolution gap to understand the heterogeneity in airway remodeling in both the large and small airways and vessels within the asthmatic lung.

### Treatment of airway remodeling

The current 2021 guidelines from the Global Initiative for Asthma (GINA) aim to control symptoms and minimize the risk of exacerbations and persistent airflow limitation (GINA Asthma, 2012). As per the 2021 GINA guidelines, the first line of recommended pharmacological treatments are inhaled low-dose corticosteroids (ICS) to suppress the inflammatory response within the airways, and bronchodilators to relax the smooth muscles during bronchoconstriction (Barnes, 2010). However, there are no current asthma pharmacological treatments that are used primarily to target airway remodeling (Berair and Brightling, 2014). Bronchial thermoplasty is a non-pharmacological approach that uses controlled thermal energy to reduce smooth muscle mass in severe uncontrolled asthma (Dombret et al., 2014), and thus is the only asthma therapy that directly targets airway remodeling. Recent follow-up studies have shown that ACQ scores and airway resistance can be sustained up to 10 years after treatment with bronchial thermoplasty, however, there were no significant changes in FEV_1_ and patients that undergo this procedure have an increased risk for bronchiectasis (Langton et al., 2018; Langton et al., 2020; Chaudhuri et al., 2021). This could be in part due to the treatment only targeting the large central airways, and not the small conducting airways which are the site of airway narrowing and closure in asthma (Donovan et al., 2018). Langton et al. also demonstrated that following bronchial thermoplasty treatment, not all airways were dilated uniformly (Langton et al., 2021). Thus, while bronchial thermoplasty treatment can be a useful approach for some patients with severe uncontrolled asthma, it has limitations to modify airway remodeling across the entire lung structure and the spectrum of disease severity and endotypes. Below we outline the clinical studies to date, which have investigated the effect of pharmacological treatment on features of airway remodeling.

#### Inhaled corticosteroids (ICS)

It was first shown in a double-blind randomized controlled trial, that 12 months of fluticasone propionate treatment decreases reticular basement membrane thickness, though there were no effects on collagen I and III deposition (Ward et al., 2002). ICS treatment has also been shown to decrease collagen type III subepithelial deposition in bronchial biopsies (Mattos et al., 2002). However, given that multiple other longitudinal studies show ICS treatment does not affect reticular basement membrane thickness, more research is needed to determine why there is heterogeneity in ECM remodeling to ICS treatment (Jeffery et al., 1992; Boulet et al., 2000; Bergeron et al., 2005). This will be particularly important as the new 2021 GINA guidelines for asthma recommend low-dose ICS along with the use of long-acting bronchodilators.

With regards to airway epithelial damage, Dorsheid et al. showed that treatment with dexamethasone, beclomethasone, budesonide, and triamcinolone on primary airway epithelial cells induced apoptosis (Dorscheid et al., 2001). Several studies have also demonstrated that when ciliated cells are exposed to ICS, their adhesion to the basal lamina is weakened, leading to epithelial shedding (Shebani et al., 2005; Trautmann et al., 2005; Uhlík et al., 2007). In a study investigating the effects of inhalation from a single dose of beclomethasone in a rabbit model, Uhlík et al. showed that the mucus release was accelerated from goblet cells and due to overstimulation they became degenerated and were eventually shed (Uhlík et al., 2007). However, there is some conflicting evidence of how epithelial shedding occurs or loss of barrier function may be induced by ICS use. Carayol et al. demonstrated that dexamethasone treatment was able to recover TNF-α induced reduction of adherens proteins E-cadherin, β and γ-catenin expression (Carayol et al., 2002). Further, Laitinen et al. showed that following 3 months of use of ICS, there was an increased number of ciliated cells and a reduction of goblet cells in biopsies from asthmatic patients (Laitinen et al., 1985). This indicates that the effect of ICS treatment on epithelial damage is time dependent. Indeed, in a longitudinal study looking at bronchial mucosal biopsies from before and after 10 years of ICS treatment, Lundgren et al. showed that though the number of inflammatory cells was reduced post-treatment, only partial recovery of epithelial damage was observed (Lundgren et al., 1988).

There are limited studies on how ICS use affects the pulmonary vasculature in asthma. Notably, Hoshino et al. showed that 6 months of treatment with inhaled beclomethasone dipropionate lead to a significant reduction in vessel number and vascularity within the lamina propria (Hoshino et al., 2001b). However, in a shorter study that lasted 6 weeks of both low-dose and high-dose inhaled fluticasone propionate, only patients administered a high dose of ICS had a decreased number of vessels and an overall vascular area within the airway wall (Chetta et al., 2003).

When assessing the effects of long-term use of ICS at high doses on airway remodeling, it is important to note the side effects of ICS which include impaired growth in children, decreased bone mineral density, and cataracts (Dahl, 2006). As well, longitudinal studies have demonstrated that patients with asthma who are taking ICS and/or bronchodilators continue to have fixed airflow obstruction (Phelan et al., 2002; Sears et al., 2003). Thus, while the use of ICS to manage airway inflammation will be important for sustained long-acting bronchodilator use ICS treatment of airway remodeling may not be feasible, and targeted therapeutics to modulate airway remodeling will be important to improve lung function.

#### Leukotriene modifiers

In asthma, leukotriene receptor antagonists including zafirlukast and montelukast have been shown to decrease inflammation, improve airway hyperresponsiveness and reduce exacerbations (Horwitz et al., 1998; Montuschi, 2010). Most of the research investigating how leukotriene modifiers modulate airway remodeling has been performed in animal models. In 1993, Wang et al. demonstrated, in ovalbumin (OVA)-sensitized rats, that leukotriene D4 decreased smooth muscle mass in the large airways, as well as airway hypersensitivity. In 2005, Henderson et al. showed that zileuton, a leukotriene synthesis inhibitor, was able to reduce smooth muscle mass, as well as decrease the thickness of the basement membrane and the number of blood vessels in OVA-sensitive mice (Chen et al., 2013). More recently, in OVA-sensitive mice, pranlukast, a leukotriene receptor antagonist, has been shown to decrease several features of airway remodeling, including goblet cell hyperplasia, and collagen deposition by inhibiting TGF-β signaling (Hur et al., 2018). However, the challenge is converting these observations in preclinical models to humans as many induced airway remodeling features are spontaneously reversible in murine models. Future work is required in preclinical human cell models or clinical trials to determine if leukotriene modifiers can be used to modify airway remodeling in asthmatic patients.

#### Biologics

Humanized antibodies, are immune-modulating drugs, also known as biologics, which are used to target Th2-high inflammation in severe asthma. Omalizumab, the first available humanized antibody for severe asthma inhibits IgE binding to the FcεR receptor present on eosinophils, mast cells, basophils, and dendritic cells (Kumar and Zito, 2022). In clinical trials, it has been shown that omalizumab can reduce total airway wall thickness, the thickness of the reticular basement membrane, and in particular, fibronectin deposition in severe asthmatic airways (Hoshino and Ohtawa, 2012; Riccio et al., 2012; Zastrzeżyńska et al., 2020). However, these reductions were modest with only a 5% decrease in the percent wall area. Mepolizumab is a humanized antibody that selectively binds to IL-5, the primary pro-inflammatory mediator responsible for eosinophilic formation and maturation, elevated in Th2-high asthma endotypes (Cada et al., 2016). Flood-Page et al. have shown that mepolizumab treatment in mild atopic asthmatics (*n* = 24) results in a reduction of tenascin and lumican, two reticular basement membrane proteins that are upregulated in asthma. The mechanism in which mepolizumab may be modulating the ECM is not clear, though it has been proposed to be due to the reduction of eosinophils producing TGF-β1, reducing myofibroblasts and ECM protein expression (Flood-Page et al., 2003). Benralizumab is another humanized antibody that also targets IL-5 activation (Pelaia et al., 2018). Chachi et al. used a novel computational modelling approach to predict the impact of benralizumab based on eosinophilic count from bronchial biopsies, and predicted using their virtual patient model that benralizumab may reduce ASM however no *in vivo* data exist (Chachi et al., 2019).

#### Macrolides

Macrolides are a class of antibiotics that have anti-bacterial, anti-viral, and anti-inflammatory properties which can reduce exacerbations in severe asthma, typically in oral formulation (Brusselle et al., 2013; Wang et al., 2020). Azithromycin is a macrolide that has been shown to reduce ASM cell viability, leading to apoptotic cell death and reduced proliferation (Stamatiou et al., 2009; Stamatiou et al., 2010). In the past few years, it has been shown in both naïve and allergic mice models, azithromycin not only decreases airway remodeling but also ASM thickness in both proximal and distal airways (Kang et al., 2016; Donovan et al., 2020). Another macrolide that has been explored is roxithromycin, which has an autophagic effect on neutrophils and suppresses the production of IL-8, IL-6, and GM-CSF in airway epithelial cells (Kawasaki et al., 1998). Additionally, roxithromycin has been shown to inhibit ASM cell proliferation by suppressing vascular endothelial growth factor (VEGF), and extracellular signal-regulated kinase (ERK) (Pei et al., 2016). In 1994, Shimizu et al. assessed the effectiveness of roxithromycin for improving asthma symptoms and showed that it reduces bronchial hyperresponsiveness in children with asthma (Shimizu et al., 1994). Macrolides provide promising immunomodulatory effects however, their beneficial effects on airway remodeling *in vivo* have yet to be confirmed.

#### Mucolytics

Dating back to the 1960s, mucolytics were originally pursued for their ability to clear mucus plugs in muco-obstructive diseases such cystic fibrosis (CF) and chronic obstructive pulmonary disease (COPD) (Ehre et al., 2019). Mucolytics decrease mucus viscosity, enabling greater clearance, whereas expectorants are used to induce the expulsion of mucus from the respiratory tract. Small studies have shown the potential for use of inhaled N-acetylcysteine (NAC), a mucolytic and antioxidant agent (Millman et al., 1985). However, a randomized clinical trial demonstrated that NAC did not have a significant effect on improving asthma exacerbations (Aliyali et al., 2010). Yet, NAC has been shown to improve airway inflammation by modulating claudin 18 (tight junctional protein) expression (Lee et al., 2020). Further studies are needed to determine if NAC is effective for the treatment of asthma mucus plugging. Additionally, existing mucolytics including nacystelyn and methylcysteine hydrochloride, have been administered to CF patients however no studies, to our knowledge, exist for its use in asthma (Rogers, 2002). More recently, other novel mucolytics have been explored for the treatment of mucus plugging in asthma. Tris (2-carboxyethyl) phosphine (TCEP) can rapidly disrupt mucin disulfide bonds, and Morgan et al., have demonstrated its ability to reverse mucus plugging in an allergic mouse model (Morgan et al., 2021). Much more work is needed to provide effective mucolytic therapeutics to improve mucus plug clearance in asthma.

#### Other therapeutics

More recently, pharmacological targeting of the ASM has been of particular interest. In 2015, Girodet et al. showed in a large double-blinded, randomised study that after 12 months of treatment with gallopamil (an L-type calcium channel blocker normally used to treat coronary heart disease), there was an 18% reduction of ASM thickness in severe asthma at the fourth generation, measured by CT, and additionally, that there was a significant decrease in ASM cell proliferation (Girodet et al., 2015). The following year, Gonem et al. began investigating the effects of fevipiprant, a prostaglandin D_2_ antagonist in a phase 2 randomized, placebo-controlled trial, which reduced eosinophilic inflammation in the airways by blocking PGD_2_-driven release of Th2 cytokines and improved lung function (Gonem et al., 2016). Then, analysis of bronchial biopsies from the same patients with asthma in the previous study revealed that fevipiprant reduced airway smooth muscle mass in patients with asthma, likely by decreasing airway eosinophilia and decreasing recruitment of myofibroblasts and fibrocytes (Saunders et al., 2019). Though this drug initially seemed promising for the treatment of persistent severe asthma, in a phase 3 randomised, double-blind, placebo-controlled trial, there was found no significant improvement in lung function (Castro et al., 2021). Finally, the use of thiazolidinediones, oral insulin-sensitizing medications typically used to treat hyperglycemia and type 2 diabetes mellitus, has been examined for asthma treatment. A study by Ward et al. has shown that rosiglitazone has anti-proliferative effects on ASM cells in culture (Ward et al., 2004). Though there are some modest beneficial effects on allergen response, based on the results from initial clinical trials, thiazolidinediones are unlikely to have therapeutic benefit for airway remodeling (Richards et al., 2010; Sandhu et al., 2012).

Thus, much further work is required to assess the responses of current pharmacological agents for asthma on airway remodeling. However, as it has been highlighted by earlier studies, the concept that inflammation and airway remodeling are tied may not be correct and targeted therapies focused on airway remodeling features may be required.

## Summary

This review article provides an overview of the existing knowledge on airway remodeling features observed in asthma, including epithelial damage, mucus cell metaplasia, ECM remodeling in both the airways and vessels, angiogenesis, and increased smooth muscle mass. While such studies have provided extensive knowledge on different aspects of airway remodeling, they have relied on biopsy sampling or autopsy lungs which have limitations. Biopsy samples are limited in the airway wall structures that can be sampled and the tissue artefacts that can be induced when crunched by the biopsy forceps. Autopsy studies on lungs from fatal asthmatics offer a unique opportunity to assess the heterogeneity of airway remodeling within a lung, but it does not provide the opportunity to assess the spectrum of the asthma syndrome. In this review, we highlight the potential of *in vivo* imaging tools such as CT and MRI that are enabling measures of the airway wall *in vivo* and longitudinally in research studies. Importantly, such volumetric imaging tools provide the opportunity to assess the heterogeneity of airway remodeling within the whole lung and has led to the novel identification of heterogenous gas trapping and mucus plugging as important predictors of patient outcomes. However, still more work needs to be done to now assess how these radiological features can be understood pathologically and then translated to therapeutic targets. Lastly, we summarize the current knowledge of modification of airway remodeling with available asthma therapeutics to highlight the need for future studies that could now use *in vivo* imaging tools to assess airway remodeling outcomes with therapeutics.
